# New-York Swindlers

**Published:** 1871-04

**Authors:** 


### NEW-YORK SWINDLERS.

Farmers and others are frequently in receipt of circulars by mail, in which they are solicited to invest in various speculations, by means of which they are promised large profits. They doubtless wonder how these rogues come in possession of their names—perhaps we can enlighten them: Several days ago the BISTOURY was in receipt of a letter asking whether we were in possession of any Directory giving the names and postoffice address of all farmers and others in our county, or whether we could furnish a manuscript list of such persons, and if so, the author of the letter would pay liberally therefor,when the same was received by Wm.Hamilton, 22 Ann St., at the office of the Farmers' Gazette. As the letter is lithographed, doubtless thousands of editors and others, have received copies, and many have replied, sending Mr. Hamilton the desired names, and so affording him an opportunity for sending some swindling circular to the farmers throughout the country.

Hamilton's circular would lead a thoughtless person to suppose that he desired to send copies of the "Farmers' Gazette" to our farming population, but when it is known that a strict search for a periodical of that name, in "Rowell's Newspaper Directory," reveals the fact that no such journal is published in New York, the inference is that Mr. William Hamilton of 22 Ann St., is one of the many swindlers of that city, who is a dealer in "queer," counterfeit money, "Oride or Eagle Watches," or some other confounded swindle of the sort.

One of the most profitable of the New York swindles is this "Queer" or counterfeit money business. Quite a large number of Sharpers have made vast fortunes by it, their incomes having been from one to fifteen hundred dollars daily. Having secured the names of parties all over the land, in the manner that Wm. Hamilton secures his, they address circulars to them, offering to forward packages of counterfeit money, in amounts of $50 to $5,000, upon receipt of from ten to two hundred dollars in genuine bills. They guarantee the imitation bills to be so perfectly executed as to defy detection, and urge the receiver of the circular to order a test package by express. If the money is sent, the sharper pockets it, and never sends anything to his victim, who, of course, cannot complain of sharper, for if he does he exposes himself as a rouge desirous of dealing in spurious money.—Isn't that a sharp dodge? Thousands of innocent idlers and loafing vagabonds throughout the land, bite at this tempting bait and become deservedly swindled. Those who have secured immense fortunes at this business call themselves James A. Holt, Fulton st.; Wm. E. Anthony, Broadway; J. D. Terhune & Co., Broadway; Thomas G. Allison, William st.; Francis Ogden, Maiden Lane; Wm. H. Hammond; Wogan & Co.; A. J. Hitchcock & Co.; J. R. Lee & Co., and Holland & Co. The last four mentioned firms being one and the same man, whose real name is Daily, and who resides in an elegant brown-stone dwelling in Brooklyn.

Another class of Swindlers are those who address circulars to people offering to make them agents for their "Eagle" or "Oride" Watches, which "are perfect imitations of 18 karat gold watches, which will never tarnish, are good time-keepers, and are warranted in every particular to be equal to the best gold watches."—These they offer to send you—if you agree to become an agent for their sale—at the very low figure of ten or fifteen dollars, so that you can use it for a *sample watch.* If you send your ten dollars you will receive an article which cost originally about one dollar, and is as worthless as the firm from whom you seek redress; you are badly swindled, and there is no help for you. "The Eagle Watch Co." of 148 Fulton st., is a swindling concern of this character.—They advertise extensively—never pay for their advertising—cannot be found at their advertised place of business, and swindle every one who comes in contact with them.

The Divorce Lawyers of New York, whose cards can be found in a majority of the country newspapers, (and which are never paid for, unless in advance,) are another set of sharpers.—They advertise to "procure legal divorces in all the States, without publicity; desertion, drunkenness, non-support, &c., sufficient cause."—Their manner of doing business we have reported elsewhere. Suffice it to say, their divorces are *not* legal, and any man or woman holding one and re-marrying, under the supposition that they *are* genuine, are guilty of bigamy, and can be punished for the crime to the full extent of our laws. M. House has been doing a lucrative business in this direction, but has suspended operations for the present, having accepted quarters in one of our State Prisons for the next seven years. Moore & Richardson, of 180 Broadway, are the new operators of the fraudulent divorce business. We can assure publishers that if they accept their proposition to publish their card and send bill and copy of paper containing said card, that they need not worry about not receiving the promised remittance—it will never come. And those parties who hold their divorces should not be in a hurry to secure another wife, as by the laws of our land *one* wife is all a fellow is allowed, and in most instances will be found sufficient for the purposes intended.

